# A Meta-Analysis and Critical Review of Prospective Memory in Autism Spectrum Disorder

**DOI:** 10.1007/s10803-016-2987-y

**Published:** 2017-01-23

**Authors:** Julia Landsiedel, David M. Williams, Kirsten Abbot-Smith

**Affiliations:** School of Psychology, Keynes College, University of Kent, Canterbury, CT2 7NP UK

**Keywords:** Autism spectrum disorder, Event-based prospective memory, Time-based prospective memory, Meta-analysis, Review, Memory, Executive functioning

## Abstract

Prospective memory (PM) is the ability to remember to carry out a planned intention at an appropriate moment in the future. Research on PM in ASD has produced mixed results. We aimed to establish the extent to which two types of PM (event-based/time-based) are impaired in ASD. In part 1, a meta-analysis of all existing studies indicates a large impairment of time-based, but only a small impairment of event-based PM in ASD. In Part 2, a critical review concludes that time-based PM appears diminished in ASD, in line with the meta-analysis, but that caution should be taken when interpreting event-based PM findings, given potential methodological limitations of several studies. Clinical implications and directions for future research are discussed.

## Introduction

### Prospective Memory

Prospective memory (PM) is the ability to remember to carry out planned actions at the appropriate point in the future (McDaniel and Einstein 2007). Everyday examples of PM tasks include remembering to stop at the supermarket to buy milk on the way home from work, remembering to call somebody on their birthday, or remembering to turn off the bath taps before the bath overflows. McDaniel and Einstein (2007) have outlined the core characteristics of a PM task as follows. First, there must be a consciously formed intention or plan that should be carried out *in the future*. That is, there should be a delay between formation of an intention to act and the execution of that intention; if there was no delay, then the task would be more akin to a vigilance/monitoring task than a PM task, because the intention can be held in short-term/working memory for the entire period between formation and execution (Graf and Uttl 2001). Second, the PM task has to be embedded in an *ongoing activity* that requires attentional resources. Thus, a person needs to consciously interrupt the ongoing task to perform their intended action for the task to be considered a measure of PM.

In experimental studies, researchers commonly distinguish between event-based and time-based PM (Einstein and McDaniel 1990). Event-based PM involves carrying out an intention upon the occurrence of a particular event (e.g., taking the cake out of the oven when the timer goes off; taking medication after breakfast). Time-based PM involves carrying out an intention at a particular future time point (e.g., call somebody at 3 p.m.; take medication at 1 p.m.). In experimental measures of PM (time- or event-based), an ongoing task/activity might be a lexical decision task (e.g., deciding whether items that appear on-screen are words or nonwords). In an event-based task with this ongoing activity, the PM instruction might be to “press the space bar when the item ‘Dog’ appears on-screen”. In this example, the appearance of the word “Dog” represents the event that should be responded to in accordance with the PM instruction. In a time-based task with this ongoing activity, the PM instruction might be to “press the space bar at exactly 2 min intervals throughout the task”. Additionally, in time-based PM tasks, participants need to monitor time during the task in order to carry out the PM instruction. Usually, in computer-based tasks, participants can press a pre-specified keyboard key to display a clock, which remains on screen for a short period. For both event- and time-based PM tasks, performance is usually measured by: (a) the proportion of correct responses in the ongoing task (e.g., the proportion of items correctly classified as words/nonwords in the lexical decision task; *ongoing task performance*/*accuracy*), and (b) the proportion of PM failures (the proportion of occasions that participants did not carry out the PM instruction when they should have; *PM task performance*/*accuracy*). Additionally, the frequency (total number) and distribution of clock checks is another measure that is usually taken in time-based PM tasks. An adaptive time-monitoring strategy would mean that a participant only makes a few clock checks at the beginning of the task establishing a feel for the passage of time (e.g., five checks within the first minute of the task), but increasingly checks the clock more frequently closer to target time (e.g., five checks within the last 20 s before the target time) (e.g., Mäntylä et al. 2007).

Evidence for the distinction between time-based and event-based PM comes from (a) neuroimaging and lesion studies, which report that distinct sub-regions of the rostral prefrontal cortex underpin time-based vs. event-based PM (Burgess et al. 2011) and that lesions to specific regions of the rostral prefrontal cortex impair one aspect of PM, but not the other (e.g., Volle et al. 2011); (b) studies of development, which reveal different patterns of age-related improvement (in children) and decline (in older adults) in event-based vs. time-based PM (Henry et al. 2004; Kliegel et al. 2013); and (c) neuropsychology studies that indicate possible double-dissociations between these two types of PM (Altgassen et al. 2014; Katai et al. 2003). One of the crucial differences between event-based and time-based PM is the retrieval context. In event-based tasks, the occurrence of the target event can automatically activate retrieval of one’s intention (cued retrieval of one’s intention providing one registers/perceives the event). In contrast, time-based PM tasks do not have any specific event that one needs to respond to and, thus, retrieval of one’s intention must be self-initiated, which places a high demand on executive functioning.

### Neurocognitive Underpinnings of Prospective Memory

PM requires the complex interplay of several cognitive processes, including aspects of executive functioning (Martin et al. 2003). Planning is involved during the formation and encoding of an intention (Kliegel et al. 2002), and retrospective/working memory is necessary to store the delayed intention while performing the ongoing task or filler tasks (Marsh and Hicks 1998). At the same time, attentional monitoring of the environment is required to recognise the appropriate moment to initiate the PM action (Kliegel et al. 2008). Finally, in order to successfully execute one’s intention, a person has to shift their attention away from the ongoing task, which requires cognitive flexibility and inhibitory control (Kliegel et al. 2002).

Another cognitive process which is thought to play a key role in PM is episodic future thinking (the ability to project oneself mentally into the future to imagine/pre-experience future events/states of self; Atance and O’Neill 2001). Specifically, episodic future thinking is thought to play an important role during intention formation in terms of cue-to-retrieval-context association (Brewer et al. 2011). That is, episodic future thinking might support PM retrieval by strengthening the association between PM cues and the future context that they will appear in. For example, at the stage of encoding one’s intention to visit the supermarket on the way home from work, one might imagine taking the turn at the traffic light to go the supermarket instead of heading straight home. Later, when actually at the traffic light, the similarity between the environment and one’s earlier episodic simulation may help trigger the activation of the PM action (Altgassen et al. 2015). Finally, PM may well depend to some extent on mentalising ability. Specifically, the ability to represent one’s own intentions would seem to be imperative for successful PM (e.g., Altgassen et al. 2014).

### Autism Spectrum Disorder

One neurodevelopmental disorder that is characterised by impairments of several of the aforementioned neurocognitive underpinnings of PM is autism spectrum disorder (ASD). ASD is a neurodevelopmental disorder that is diagnosed on the basis of impairments in social-communication, and a restricted, repetitive repertoire of behaviour and interests (DSM 5, American Psychiatric Association 2013; ICD-10, World Health Organisation 2006). At the cognitive level, ASD is characterised by impairments in mentalising/Theory of Mind (e.g., Happé and Frith 1995), episodic memory and future thinking (e.g., Lind et al. 2014), as well as task switching (cognitive flexibility) and planning (e.g., Williams and Jarrold 2013), and visual working memory (e.g., Kenworthy et al. 2008). Because these neurocognitive abilities are impaired in ASD and an inherent component of PM, it would clearly follow that at least some aspects of PM should be impaired in ASD.

If individuals with ASD are indeed impaired in either or both event-based and time-based PM, this would likely have serious ramifications for every-day functioning. Impairments in PM, which are a common feature of normal aging (e.g., Maylor et al. 2002), can drastically reduce an individual’s ability to live independently and maintain many activities that are often taken for granted (Mateer et al. 1996; Terry 1988). At the extreme end of possible consequences, impaired PM could lead one to forget to take medication or to take food off the stove, which might have disastrous consequences. Less dramatically, an impairment in PM would seriously hinder opportunity to maintain employment (Howlin and Moss 2012). Moreover, there are even potentially negative social consequences of a PM impairment. For example, forgetting to call a friend on his birthday, or to attend a funeral, could have a significant impact on social relations, which are already difficult for people with ASD. Therefore, it is crucial to investigate PM in ASD as PM deficits could contribute to social and behavioural impairments in ASD.

In this article, we took two approaches to explore PM research in ASD. In Part 1, we will report the results of a meta-analysis that was conducted with the aim of establishing whether or not/the extent to which PM is impaired in ASD. An initial interpretation of the meta-analytic statistics is offered in Part 1. However, as discussed at length below, the results from a meta-analysis need to be interpreted carefully in light of several methodological issues with some of the studies included. Therefore, in Part 2, we provide a detailed critical reflection on the research included in the meta-analysis, which provides the background for further reflection on the analysis in Part 1.

## Part 1: Meta-analysis of Studies of PM in ASD

### Methods

#### Sample of Studies

**Fig. 1:**
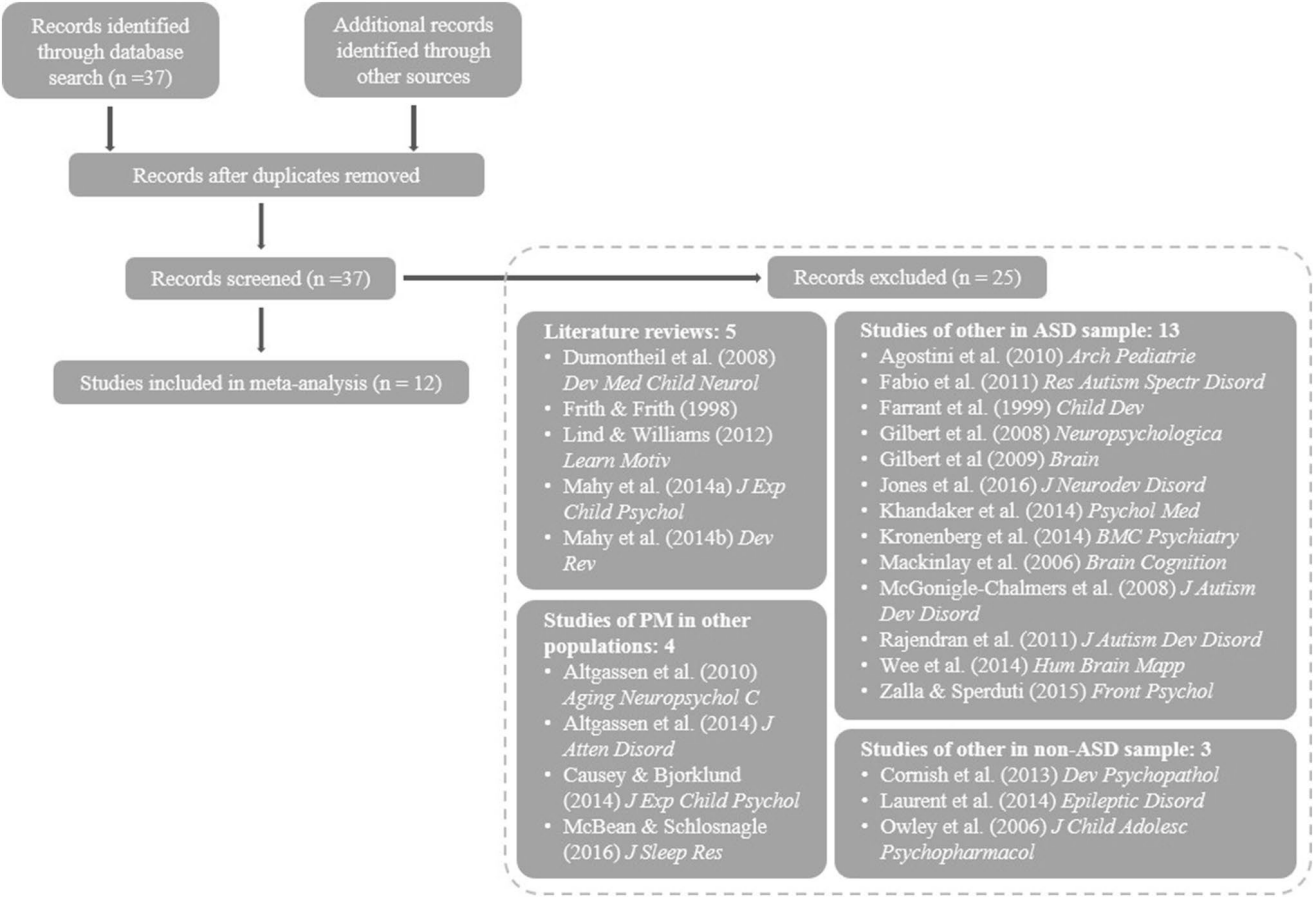
Flow-chart depicting literature search process

**Fig. 2:**
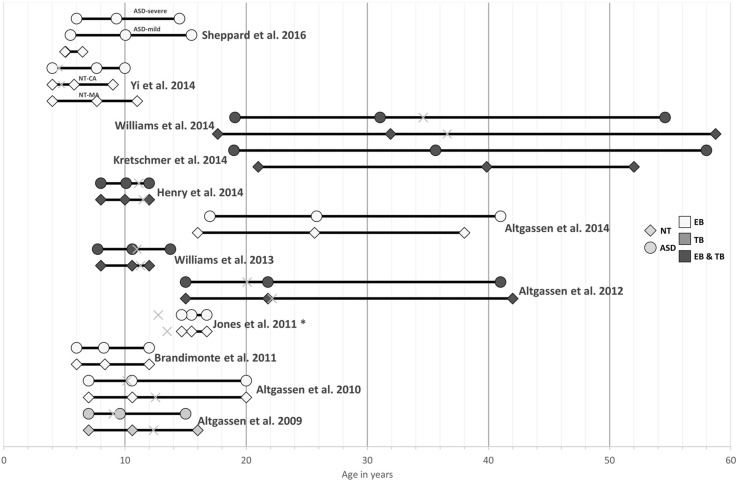
Depiction of the mean age (*middle marker*) and the minimum and maximum age range for each experimental group for all studies investigating prospective memory in ASD. Where possible we plotted the verbal mental age of each group onto the age distribution as marked with the *grey* x to illustrate the relation of chronological vs. mental age in each study. *TB* time-based PM only study, *EB* event-based PM only study, *TB* and *EB* time-based and event-based PM study, *Jones et al. (2011) did not study event-based PM per se but investigated everyday memory in ASD

A literature search (see Fig. 1) was conducted on Web of Science using the search terms “autism” AND “prospective memory” for articles published prior to May 2016 resulting in 37 articles. Of these, 13 studies with an ASD sample were excluded as they studied something other than PM. Five literature reviews were excluded that did not provide any data of their own (two of which briefly mentioned PM in ASD, two were on PM in general, and one was unrelated to PM). Another four studies were excluded as they studied PM in a population other than ASD. Finally, three studies were excluded as they were completely unrelated to ASD and PM. Hence, we identified 12 studies that had investigated PM in ASD and included these in the meta-analysis. No further studies were identified from reference lists of other included studies or by replicating our search using additional search engines (Pubmed, Google Scholar). Tables 1 and 2 summarise the included studies and give a brief overview of the experimental approach/protocols of each study. Figure 2 depicts the mean age for both ASD and the neurotypical (NT) control group, together with the overall age range and the verbal mental age of each experimental group.

**Table 1:** Overview of characteristics of time-based prospective memory studies in autism spectrum disorder

Author, year	Participants		Task characteristics		Filler tasks/delay interval	Authors concluded PM impairment in ASD group (Hedges’*g*)^a^
	Sample size (male per group)	Mean age per group (range)	Ongoing task	# of PM trials		
Altgassen et al. (2009)	*n*_*ASD*_ = 11 (n.s.) *n*_*NT*_ = 11 (n.s.)	ASD 9.6 (7–15) NT 10.6 (7–16)	Visuospatial working memory task	5 trials	Yes, ~10 min	Yes (g = −0.91)
Altgassen et al. (2012)*	*n*_*ASD*_ = 25 (20 male) *n*_*NT*_ = 25 (19 male)	ASD 21.8 (15–41) NT 21.8 (15–42)	Dresden Breakfast task	2 trials	Yes , ~15 min	Yes (g = −0.94)
Williams et al. (2013)**	*n*_*ASD*_ = 21 (20 male) *n*_*NT*_ = 21 (17 male)	ASD 10.6 (7.8–13.8) NT 10.6 (8–12)	Computer-based driving game simulation	6 trials	No	Yes (g = −0.66)
Williams et al. (2014)**	*n*_*ASD*_ = 17 (14 male) *n*_*NT*_ = 17 (14 male)	ASD 31.1 (19.1–54.6) NT 31.9 (17.7–58.8)	Word memorisation task	5 trials	No	Yes (g = −0.66)
Henry et al. (2014)*	*n*_*ASD*_ = 30 (24 male) *n*_*NT*_ = 30 (19 male)	ASD 10.1 (8–12) NT 10 (8–12)	Virtual week game, 2 within-subject condition (high vs. low task absorption)	12 trials across 3 virtual days, (2 regular/2 irregular per virtual day)	No	Yes (g = −1.02)
Kretschmer et al. (2014)*	*n*_*ASD*_ = 27 (9 male) *n*_*NT*_ = 27 (2 male)	ASD 35.6 (19–58) NT 39.9 (21–52)	Virtual week game, 2 between-subject encoding conditions (implementation intentions vs. standard)	12 trials across 3 virtual days, (2 regular/2 irregular per virtual day)	No	Yes (g = −1.01)

*n.s*. not specified

*Time- and event-based PM task within the same condition

**Time- and event-based PM task in separate conditions

^a^Effect sizes represent the standardised bias-corrected mean difference Hedges’*g* (calculation according to Lipsey and Wilson 2001)

**Table 2:** Overview of event-based prospective memory studies in autism

Author, year	Participants		Task characteristics				Filler task/delay interval	Authors concluded PM impairment in ASD group (Hedges’*g*)^a^
	Sample size (male per group)	Mean age (range) per group	Ongoing task	# of PM trials	# of PM cues	Focality of PM cue		
Altgassen et al. (2010)	*n*_*ASD*_ = 19 (18 male) *n*_*NT*_ = 19 (16 male)	ASD 10.6 (7–20) NT 10.6 (7–20)	Visuospatial working memory task	5 trials	1	Non-focal	No	No (g = −0.25)
Brandimonte et al. (2011)	*n*_*ASD*_ = 30 (21 male) *n*_*NT*_ = 30 (21 male)	ASD 8.25 (6–12) NT 8.33 (ns.)	Categorisation of pictorial images	8 trials	2	Focal	No	Yes (g = −0.96, post-hoc test, interaction not significant)
Jones et al. (2011)	*n*_*ASD*_ = 94 (85 male) *n*_*NT*_ = 55 (53 male)	ASD 15.5 (14.7–16.8) NT 15.5 (ns.)	Rivermead behavioural memory test	3 trials	1 per task	Focal	No	Yes (g = −0.41)
Altgassen et al. (2012)*	*n*_*ASD*_ = 25 (20 male) *n*_*NT*_ = 25 (19 male)	ASD 21.8 (15–41) NT 21.8 (15–42)	Dresden Breakfast task, Red Pencil Task	2 trials for each task	2 and 1	Focal	Yes	Yes (Breakfast task: g = −0.70, red pencil task: g = −0.76)
Williams et al. (2013)**	*n*_*ASD*_ = 21 (20 male) *n*_*NT*_ = 21 (17 male)	ASD 10.6 (7.8–13.8) NT 10.6 (8–12)	Computer-based driving game simulation	6 trials	1	Focal	No	No (g = 0.17)
Williams et al. (2014)**	*n*_*ASD*_ = 17 (14 male) *n*_*NT*_ = 17 (14 male)	ASD 31.1 (19.1–54.6) NT 31.9 (17.7–58.8)	Word memorisation task	4 trials	1	Non-focal	No	No (g = 0.42)
Yi et al. (2014)	*n*_*ASD*_ = 25 (19 male) *n*_*NT*−*MA*_ = 28 (19 male) *n*_*NT*−*CA*_ = 25 (22 male)	ASD 7.66 (4.9–10.3) NT_MA_ 5.8 (4.3–9.9) NT_CA_ 7.68 (4.6–11.2)	Naming of items on cards	5 trials	1	Focal	No	Yes (ASD vs. NT_MA_: g = −0.59, ASD vs. NT_CA_: g = −0.39)
Altgassen and Koch (2014)	*n*_*ASD*_ = 22 (20 male) *n*_*NT*_ = 22 (20 male)	ASD 25.8 (17–41) NT 25.6 (16–38)	Word categorisation task plus inhibition task	4 trials	1	Non-focal	Yes, ~10 min	No (g = −0.13)
Henry et al. (2014)*	*n*_*ASD*_ = 30 (24 male) *n*_*NT*_ = 30 (19 male)	ASD 10.1 (8–12) NT 10 (8–12)	Virtual week game 2 within-subject conditions (high vs. low task absorption) of 3 virtual days each	12 trials across 3 virtual days, (2 regular/ 2 irregular per virtual day)	4	Not clear	No	No (g = −0.10)
Kretschmer et al. (2014)*	*n*_*ASD*_ = 27 (9 male) *n*_*NT*_ = 27 (2 male)	ASD 35.6 (19–58) NT 39.9 (21–52)	Virtual week game 2 between-subject encoding conditions (implementation intentions vs. standard)	12 trials across 3 virtual days, (2 regular/2 irregular per virtual day)	4	Not clear	No	Yes (g = −0.55)
Sheppard et al. (2016)	*n*_*ASD*−*severe*_ = 14 (13 male) *n*_*ASD*−*mild*_ = 14 (14 male) *n*_*NT*_ = 26 (16 male)	ASD_severe_ 9.30 (6–14.5) ASD_mild_ 10.05 (5.5–15.5) NT 5.1 (5.05–6.5)	Interaction with a hand puppet (played by experimenter), playing a distractor game (‘Wac-a Mole’)	2 trials PM clapping task 2 trials PM feeding task 1 trial PM reward task	1 per task	Focal	Yes, between 1 and 5 min	Yes (NT vs. ASD_severe_: g = −1.43) No (NT vs. ASD_mild_: g = −0.57)

*Time- and event-based PM task within the same condition

**Time- and event-based PM task in separate conditions

^a^Effect sizes represent the standardised bias-corrected mean difference Hedges’*g* (calculation according to Lipsey and Wilson 2001); ns.: not specified

#### Meta-analytic Procedure

Meta-analytic statistics were calculated following the guidelines of Lipsey and Wilson (2001), separately for time-based PM and event-based PM studies. Effect sizes were calculated for the difference in prospective memory performance between ASD and NT participants. The effect size estimate was the bias-corrected standardised mean difference (Hedges’*g*), which corrects an overestimation bias of effect sizes in small-scale studies (Hedges 1981; Hedges and Olkin 1985). In the meta-analysis, a fixed-effects model was used to calculate the mean effect, expressing group differences in PM, weighted for sample size, and a 95% confidence interval (CI) was calculated based on its standard error (SE). The direction of the effect size was negative if performance of the ASD group was worse than the control group and effect sizes were classified according to Cohen’s (1988) criteria (0.20 is “small”, 0.50 is “medium”, and 0.80 is “large”). A z-test for the overall effect was conducted to test the significance of the mean weighted effect and a homogeneity analysis was conducted to test for homogeneity of the effect size distribution. A significant homogeneity parameter indicates that the variability of the included effect sizes is greater than to be expected from sampling error and suggests that other explanatory variables should be investigated. In this case, a conservative approach was adopted and an additional effect size estimate was calculated using the random-effects model.

#### Multiple Effect Sizes from Single Studies

To satisfy the independence assumption of meta-analyses when calculating the mean weighted effect for time- and event-based PM, respectively, each participant could contribute to only one group contrast for statistical analytic purposes. Therefore, it was not possible to include all calculable effect sizes in three of the included studies, because doing so would have violated the assumption of independence in one of the following ways: (a) multiple ASD groups but only one NT group would have meant that the NT group would be included in the meta-analysis more than once if all reported group contrasts were included (Sheppard et al. 2016); (b) multiple NT groups but only one ASD group would have meant that the ASD group would be included more than once if all reported group contrasts were included (Yi et al. 2014); or (c) multiple PM measures from the same participants would mean that each participant would be included more than once if performance on all measures was included (Altgassen et al. 2012). Furthermore, to avoid biasing the mean weighted effect, group contrasts that explored the effect of attempts to improve PM in the ASD were excluded (Kretschmer et al. 2014). Full details of the procedure for deciding which effect size should be included in the meta-analysis are reported.^1^

### Results

**Fig. 3:**
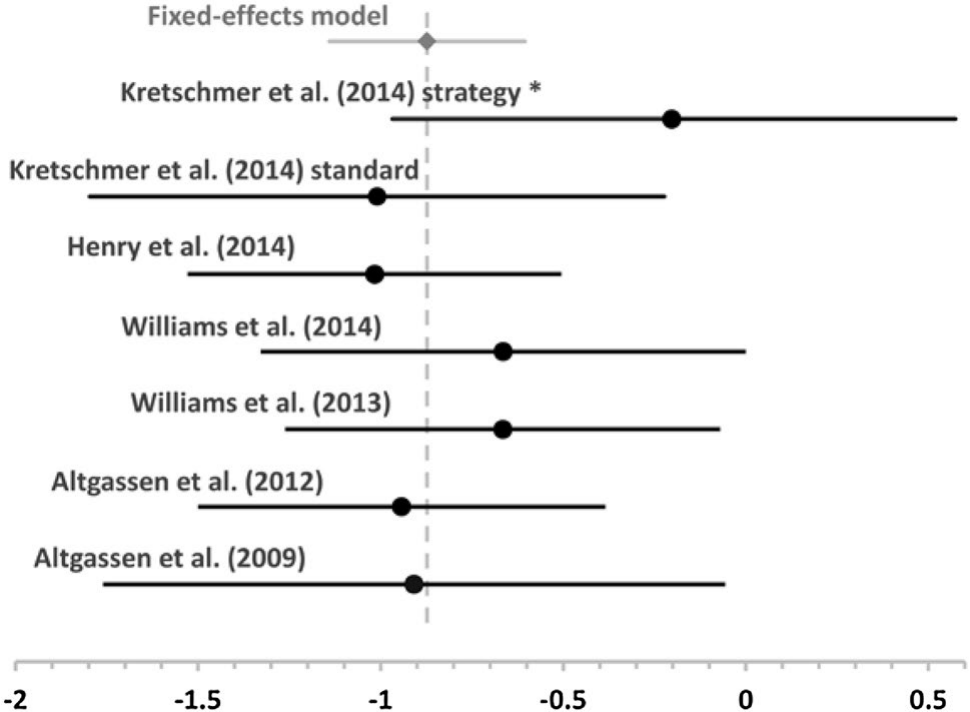
Forest plot for effect sizes and 95% confidence interval for time-based PM studies as well as the mean weighted effect and its 95% confidence interval (in *grey, diamond marker*). *Vertical grey line marks* the weighted mean effect. Studies marked with an *asterisk* were not included in the meta-analysis

For time-based PM, a total of 118 participants with ASD and 118 NT control participants from six studies were included using a fixed-effects model. The weighted effect for the between-group difference in performance was −0.87 (SE 0.14, 95% CI −1.14 to −0.60; z = 6.38, p < .001). The homogeneity test was non-significant (*Q* = 1.22, p = .94) indicating that the variance across the included effect sizes was not greater than expected by sampling error. These findings indicate a large and consistent impairment of time-based PM in ASD across studies (see Fig. 3).

**Fig. 4:**
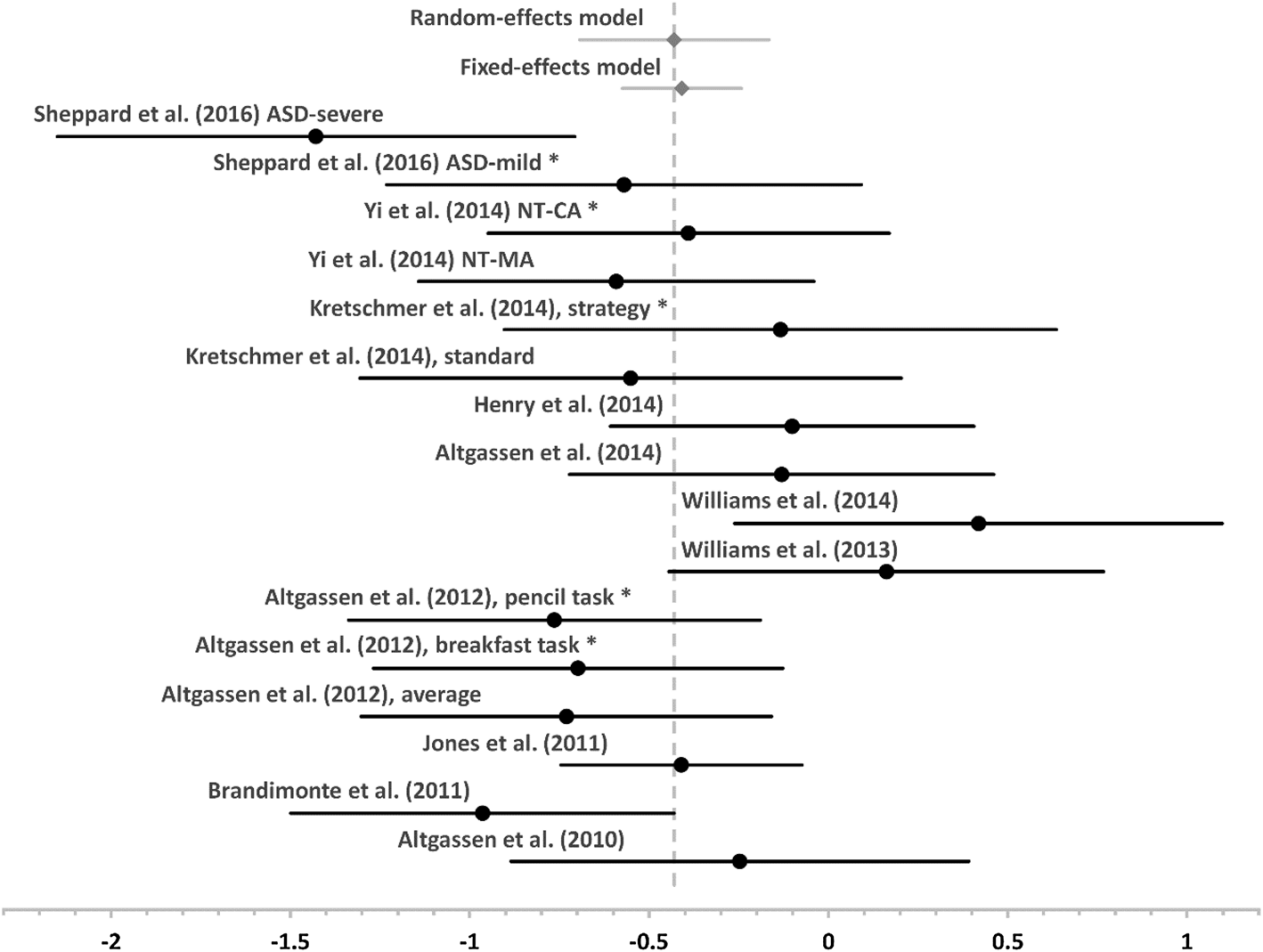
Forest plot for effect sizes and 95% confidence interval for event-based PM studies as well as the mean weighted effect and its 95% confidence interval for both fixed- and random-effects models (in *grey, diamond marker*). *Vertical grey line marks* the weighted mean effect of the random-effects model. Studies marked with an *asterisk* were not included in the meta-analysis

For event-based PM, a total of 311 participants with ASD and 287 NT control participants from 11 studies were included in the fixed-effects model. The weighted effect for the between-group difference in performance was −0.41 (SE 0.08, 95% CI −0.57 to −0.24; z = 4.83, p < .001). However, the test for homogeneity of effect sizes was highly significant (*Q* = 25.14, p = .005), which suggests that the variance among the included effect sizes was greater than expected by sampling error. Subsequently, the data was re-entered into a random-effects model that includes random effects variance (due to random differences between studies) in the weighting of the individual effect sizes in the model. This revealed a significant weighted effect of −0.43 (SE 0.13, 95% CI −0.69 to −0.17; z = 3.20, p < .01). Hence, both models indicate a small impairment of event-based PM in ASD, although the underlying effect sizes are heterogeneous across studies (see Fig. 4).

Although the results of the meta-analysis provide some indication of a relatively minor impairment in event-based PM in ASD, this stands in stark contrast to the clear and strong effect of a time-based PM impairment in individuals with this condition. Some caution might be taken when interpreting the findings with regard to event-based PM, however. Although results from the random effects model suggest that a significant weighted effect for event-based PM overall cannot be solely due to significant heterogeneity of effect-sizes across studies, the existence of such heterogeneity is nonetheless important to consider. Indeed, in Part 2, we provide a detailed critical analysis of the studies included in the meta-analysis and conclude that the meta-analytic findings of a small impairment in event-based PM derive in large part from methodological issues in some of the studies, which might render conclusions from the meta-analysis alone unreliable if they are not considered *in context*.

## Part 2: Critical Analysis of Studies of PM in ASD

### Methodological and Conceptual Issues in the Study of Prospective Memory in ASD

In order to interpret the results of case-control studies of PM ability with confidence several issues should be considered. Table 3 presents an overview of all studies included in the meta-analysis, as well as an indication of whether any of the potential methodological problems described below apply to them.

**Table 3:** Overview of included studies with regard to key methodological issues

	Matching on baseline characteristics and Cohen’s *d* for matched variables				Matching on ongoing task performance and Cohen’s *d*		Retrospective memory for PM instruction checked	Mixed PM experiment
Altgassen et al. (2009)	Yes	Age 0.36	VA 0.50	NVA 0.30	No	1.19	Not reported	No
Altgassen et al. (2010)	Partly	Age 0.01	VA 0.58	NVA 0.25	Yes	0.09	Not reported	No
Brandimonte et al. (2011)	Partly	Age 0.04	FSIQ 0.29	–	No	0.55	Yes	No
Jones et al. (2011)	Yes	Age 0.0	VIQ 0.25	PIQ 0.04	NA	–	Yes	No
Altgassen et al. (2012)	Yes	Age 0.0	VA 0.44	NVA 0.15	No	1.60	Not reported	Yes
Williams et al. (2013)	Yes	Age 0.01	VIQ 0.18	PIQ 0.18	Yes	0.5	Yes	No
Williams et al. (2014)	Yes	Age 0.07	VIQ 0.21	PIQ 0.25	Yes	0.03	Yes	No
Altgassen and Koch (2014)	Partly	Age 0.03	–	NVA 0.1	No	0.94	Not reported	No
Henry et al. (2014)	Yes	Age 0.07	VIQ 0.28	PIQ 0.24	NA	–	No	Yes
Kretschmer et al. (2014)	Yes	Age: 0.45	VA 0.25	NVA 0.03	NA	–	No	Yes
Yi et al. (2014)	No (ASD vs. NT_MA_)	Age 1.29	VA 0.13	NVA 0.63	Not reported	–	Not reported	No
	No (ASD vs. NT_CA_)	Age 0.01	**–**	NVA 0.65	Not reported	–	Not reported	No
Sheppard et al. (2016)	No (ASD_severe_ vs. NT)	Age 3.05	R 0.15 W 0.64	N 0.16	NA	–	Not reported	No
	No (ASD_mild_ vs. NT)	Age 2.76	R 0.33 W 0.07	N 0.46	NA	–	Not reported	No

*VA* verbal ability, *NVA* nonverbal ability, *VIQ* verbal IQ, *PIQ* performance IQ, *FSIQ* full scale IQ, *R* reading national curriculum point score, *W* writing national curriculum point score, *N* number national curriculum point score, *NA* not applicable

#### Group Matching Procedure

In case-control studies, experimental groups need to be carefully matched for baseline characteristics that are likely to relate to the dependent variable. Evidence suggests that PM has a clear developmental trajectory (Kliegel et al. 2008), and that both verbal (Uttl 2006; Uttl et al. 2013) and nonverbal (Cockburn and Smith 1991; Maylor 1996) intelligence are related to it. Furthermore, there is some evidence for a female advantage in PM tasks (Palermo et al. 2015). Therefore, when studying PM in ASD, it is important for groups to be matched for verbal and nonverbal ability (mental age), as well as for chronological age and gender (between-group differences should only be small in size—Cohen’s *d* < 0.50; McCartney et al. 2006). Failure to match groups for baseline characteristics can result in type I errors, because group differences in PM may result from group differences in the baseline characteristics of groups, rather than from diagnostic status (Mervis and Klein-Tasman 2004).

#### Ongoing Task Performance

A typical PM task is always embedded into an ongoing task. Thus, similar to a dual-task design, attentional and cognitive resources are divided between the completion of the ongoing task and completion of the PM action/intention. It is important that groups are matched for ongoing task performance. Otherwise, analyses of between-group differences in PM performance may be “contaminated” by the effects of between-group differences in ongoing task performance. For example, if the ASD group performs significantly *less* well than the control group(s) on the ongoing task, then poorer PM task performance in the ASD group could merely reflect the fact that ASD participants had fewer cognitive resources than control participants to devote to the PM task as a result of their difficulty with the ongoing task. Alternatively, if the ASD group performs significantly *less* well than the control group(s) on the ongoing task, but *equivalently* to the control group on the PM task, this may reflect differential allocation of cognitive resources performance in the ASD group (i.e., PM performance is being scaffolded/prioritised at the expense of ongoing task performance).

#### Retrospective Memory for the PM Instruction

PM requires an individual to encode and store an intention to act in the future, which relies on *retro*spective memory. While it is clear that retrospective and prospective memory are related, factor analytic studies (among other types of study) show that they are clearly distinguishable (Crawford et al. 2003; Maylor et al. 2002). PM failure could result either from a failure of the specific mechanisms that underpin prospective memory retrieval, *or* from a failure of retrospective memory (i.e., when the intention is not even encoded/stored properly). Given well-established difficulties with spontaneous, episodic recall in ASD (Boucher et al. 2012), it may be that people with this disorder have PM impairments purely as a result of retrospective memory difficulties. This would be important to know, of course, and would have implications for the management of PM difficulties in ASD. However, most studies have the aim of discovering whether the mechanisms that *specifically* underpin PM retrieval are impaired in ASD (i.e., whether PM is impaired over and above retrospective memory). To achieve this aim, it is necessary to assess retrospective memory for the PM instruction/action immediately after completion of the experimental PM task. If a participant cannot recall the PM action even after prompting, it shows that PM failure is a consequence of retrospective memory difficulties only. Hence, studies should exclude those participants who, after prompting, cannot remember the PM instruction/action.

#### Mixed PM Experiments

Some studies of PM use paradigms that test both time- and event-based PM within the same condition. Although this *may* be more representative of real-life PM demands, the approach suffers from difficulties that limit interpretation of results. Specifically, if individuals with ASD have reliable impairments in only one aspect of PM, then difficulties with this aspect would leave fewer cognitive resources than would otherwise be the case for the usually-undiminished aspect. Thus, the recommended approach is to investigate time- and event-based PM in separate experiments (or experimental conditions of a task) within the same sample.

Having considered these potential methodological issues, we now discuss four landmark studies that serve as a foundation for considering other studies of PM in ASD. Then, we examine further laboratory studies, as well as studies that explored PM in ASD in a more naturalistic context.

### Landmark Studies of PM in ASD

In the first study of *event-based* PM in ASD, Altgassen, Schmitz-Hübsch, and Kliegel (2010) compared 19 children/adolescents with ASD to 19 age- and ability-matched NT peers. The ongoing task tapped visuospatial working memory. In a study phase of this task, participants viewed a number of geometric shapes, and had to encode and store the configuration of shapes. After a short delay, a second set of geometric shapes appeared on the screen and participants had to decide whether the shape configuration was same or different to the first one (recognition phase). The background colour on which the shapes were presented changed randomly after each trial. For the PM component, participants were instructed to press a pre-specified keyboard key whenever they noticed a change in background colour to yellow. Participants performed the ongoing task alone for 10 trials (single-task block), followed by the PM condition (dual-task block). The results revealed no between-group differences in ongoing or PM task performance. The authors concluded that event-based PM, which depends on cued retrieval, is unimpaired in ASD.

Altgassen and colleagues were also the first to investigate *time-based* PM performance in individuals with ASD. Altgassen et al. (2009) assessed 11 children/teenagers with ASD and 11 age- and ability-matched NT control participants. The ongoing task required participants to perform a visuospatial working memory task similar to the one used by Altgassen et al. (2010). In the PM condition, participants were instructed to press a pre-specified keyboard key at 2-min intervals throughout the ongoing task. During this condition, participants could, at any time, bring up an on-screen clock that displayed the time elapsed by pressing a specified key. Importantly, the ongoing task was carried out twice - once as single-task block (ongoing-only condition), and once as dual-task block together with the PM instruction (PM condition). The ASD group performed significantly worse in the ongoing task during the PM condition, but not in the ongoing-only condition. More importantly, the results revealed significantly better PM performance, as well as a more adaptive time-monitoring curve, in NT children. Therefore, the authors concluded that the diminished PM performance in ASD might originate from difficulties with self-initiated processing, as reflected by a less-than-optimal pattern of time monitoring.

Despite some methodological concerns regarding Altgassen et al.’s (2009) study (see Williams et al. 2013), the conclusions drawn both from this study (that time-based PM is impaired in ASD) and the study by Altgassen et al. (2010) (that event-based PM is unimpaired in ASD) are supported by the results from two studies by Williams et al. (2013, 2014). Williams et al. (2013) examined both time-based and event-based PM in a sample of 21 children with ASD and 21 NT children matched on age, and IQ. Time-based vs. event-based PM were assessed separately as two within-subject conditions carried out within the context of a computer-based driving game (the ongoing task). The ongoing task required participants to collect tokens and avoid obstacles while driving down a road. For the time-based PM task, participants were told that their car had only a limited amount of fuel, which would run out after 80 s unless they remembered to refuel it. The fuel level could be monitored at any time by pressing a particular keyboard key, which caused a fuel gauge to be displayed on screen temporarily. Importantly refuelling was only possible after the fuel level dropped to a critical level (between 60 and 80 s). For the event-based PM task participants had to press a specific keyboard key whenever they passed a truck. Results revealed a significant Group (ASD/control) × Condition (event-based/time-based) interaction effect on PM task performance, reflecting preserved event-based PM performance but impaired time-based PM in the ASD group. More adaptive time monitoring (i.e. a greater number of fuel checks prior to the period where refuelling was possible) was related to fewer time-based PM failures in both groups. Importantly, groups did not differ in ongoing task performance, nor time-monitoring frequency or pattern.

The results of Williams et al. (2013) were replicated precisely in a subsequent study by Williams et al. (2014) of 17 *adults* with ASD and 17 age-, and verbal and performance IQ-matched NT adults. The same ongoing task (which tapped verbal short-term memory) was used for both PM conditions, which were carried out separately in counter-balanced order. In the ongoing task, participants studied sequences of seven words across 40 trials. After each study trial, a test list of seven words appeared on-screen and participants had to decide whether all seven had been present on the immediately-preceding study list. The event-based PM instruction required participants to press a specific key when one of the test list words represented a musical instrument. For the time-based PM task, participants had to press a specific key every two minutes throughout the ongoing task. Participants could bring up a clock displaying the elapsed time via key press. Williams et al. (2014) found a significant Group × Condition interaction, reflecting diminished time-based but spared event-based PM performance in the ASD group. Again, groups did not differ in ongoing task performance or time-monitoring frequency.

In summary, there seems to be a consistent pattern emerging from these initial studies suggesting that time-based PM is impaired, but event-based PM is unimpaired, in ASD. To explore this pattern further we first review four additional studies, which have investigated event-based PM *separately* from time-based PM, followed by a review of more naturalistic studies of time- and/or event-based PM.

### Is ASD Characterised by Truly Unimpaired Event-Based PM?

Firstly, Yi et al. (2014) studied the role of executive functioning in event-based PM in a sample of 25 children with ASD and two NT comparison groups. One comparison group was reported to be matched with the ASD group for chronological age (NT_CA_, *n* = 25), whereas the other comparison group was reported to be matched with the ASD group for verbal *mental age* and nonverbal *IQ*, (NT_MA_, *n* = 28). In Yi et al.’s paradigm, the ongoing task involved naming pictorial items on a series of cards. The PM task was to hand the experimenter a “target” card that had a red heart-shaped sticker on it. The ASD group performed significantly worse than both comparison groups on the PM task. Although this is an interesting study, there are two potential methodological issues with Yi et al.’s procedure that might lead to caution when interpreting the results. Firstly, ongoing task performance was not reported and memory for the PM task instruction was not checked. It is not clear whether participants with ASD either noticed the target sticker on the relevant cards or even that they encoded the instruction to hand the cards with such a sticker to the experimenter. Secondly, based on the data provided, we believe that the groups were not equated on baseline cognitive abilities. The NT_CA_ group was not equated for verbal mental age or nonverbal IQ, whereas the NT_MA_ group was not matched on nonverbal IQ or chronological age. Although the authors stated that the NT_MA_ group was matched with the ASD group for nonverbal IQ this was not accurate. Rather, the groups were matched for *raw* scores on the Combined Raven’s Matrices test of nonverbal ability, but not for the *standardised* scores (i.e., not for nonverbal IQ). Crucially, the standardised score among ASD participants was an average of 11 or 12 points below that of the comparison groups and was in the “below average” range (M = 79.17; SD = 21.83). In general, it is not clear why Yi et al. adopted this matching strategy. Ideally, case and control groups are matched for age and IQ, as other studies have shown is possible when investigating PM in ASD (see Williams et al. 2014).

The second such study also examined solely event-based PM in a similar age group. Brandimonte et al. (2011) studied event-based PM and response inhibition among 30 primary school-aged children with ASD, as well as 30 age, and full-scale IQ-matched and NT comparison participants. All participants completed a computerised *ongoing* task that involved sorting pictorial items into one of two categories (food and animals) via key press. The participants of each experimental group (*n* = 30 per group) were assigned to *one* of three between-subject conditions of the ongoing task. In a “PM condition”, *n* = 10 ASD and *n* = 10 NT participants completed the ongoing task as described, but had the additional requirement to press a particular keyboard key whenever pre-specified images appeared. In other words, participants had to encode and retain a PM instruction while completing the ongoing categorisation task (hence, this was a standard PM task). In a “response inhibition” condition, *n* = 10 ASD and *n* = 10 NT participants completed the ongoing task, but had the additional requirement to *not respond* (i.e., not make a categorisation judgement) when pre-specified images appeared. The requirements of this condition resemble a classic “Go/No-Go” task (Verbruggen and Logan 2008). Finally, in an “ongoing-only” condition, *n* = 10 ASD and *n* = 10 NT participants completed the ongoing task as described, but with no additional secondary requirements. This ongoing-only condition could be considered a control condition to establish how able participants with ASD are to perform the ongoing task independent of their PM or response inhibition skills. Brandimonte et al. (2011) performed two ANOVAs on their data. First, a 2 (Group: ASD/NT) × 3 (Condition: ongoing/PM/response inhibition condition) was conducted with ongoing task accuracy (percentage of correctly categorised images) as the dependent variable. There was a significant main effect of group, indicating that the comparison groups performed better than the ASD groups ($\eta _{p}^{2}$ = 0.07, p = .05) in the image categorisation task.

Second, a 2 (Group: ASD/NT) × 2 (Condition: PM/response inhibition) ANOVA was conducted using secondary task accuracy (percentage of PM successes/percentage of responses correctly inhibited) as the dependent variable. In this ANOVA, there was a significant main effect of group ($\eta _{p}^{2}$ = 0.10, p < .05) indicating better performance in both secondary tasks (PM *and* response inhibition) in the comparison group than in the ASD group. Crucially, the Group × Condition interaction was reported as non-significant and associated with a negligible effect size ($\eta _{p}^{2}$ = 0.02, p = not reported). Despite the minimal interaction effect, Brandimonte et al. (2011) nonetheless broke it down using planned comparisons. These comparisons suggested that the ASD group was less accurate ($\eta _{p}^{2}$ = 0.11, p < .05) and responded slower ($\eta _{p}^{2}$ = 0.42, p < .01) than the NT group only in the PM task, whereas no between-group differences were evident in the Go/No-Go task (no effect sizes reported). Therefore, the authors concluded that individuals with ASD have a deficit in event-based PM, but are less affected in their response inhibition. However, there are some methodological issues that need to be addressed. First, breaking down non-significant interaction effects is problematic both statistically and conceptually, and may lead to erroneous conclusions being drawn (see Gelman and Stern 2006). Second, groups were clearly not equated for ongoing task performance and the difficulties with this aspect of the experiment for participants with ASD could account for their difficulties with the secondary tasks (i.e., PM and response inhibition). Finally, although Brandimonte et al. (2011) matched the participant groups for age and FSIQ overall, it is not clear that this was the case for *each* of the between-subjects experimental conditions (i.e. PM, inhibitory control and the ongoing-task-only conditions). It is quite possible (but not reported in the paper) that diagnostic groups for each between-group condition were not comparable on these baseline variables, which could have generated artificial group differences in PM performance.

Third and most recently, Sheppard et al. (2016) investigated the effect of autism symptom severity on event-based PM in 28 children with ASD and 26 NT controls. Severity of autism symptoms in the ASD group was assessed with the Child Autism Rating Scale (Schopler et al. 1980). Scores were used to divide the ASD sample into “severe ASD” and “mild ASD” groups (*n* = 14 each). In a game-like procedure participants interacted with a hand-puppet wolf (Wally, acted out by the experimenter) playing a distractor task game alternating with the completion of three different PM tasks, and a retrospective memory task. Thus, there was no ongoing task as such. The PM tasks required the child to remember to (a) clap when they heard music being played (two trials, *PM clapping task*); (b) remove a toy food item out of Wally’s view because he could not eat them (two trials, *PM feeding task*); and (c) collect their reward at the end of the session (signalled by the experimenter saying “The games are now finished, time to go back to class.”) (*PM reward task*). For the PM clapping and the PM reward tasks, participants were prompted once *at the end of* each trial if they did not spontaneously remember the PM activity (children were asked: “Can you hear the music?” for the PM clapping task, and “Have you forgotten anything?” for the PM reward task). Collapsed across all three PM tasks, a significant main effect of group ($\eta _{p}^{2}$ = 0.13) was found. Post-hoc tests revealed that this was driven by the significant difference between the severe ASD and the NT group (d = 1.39). When examining the results separately, a similar pattern was found for the PM feeding task ($\eta _{p}^{2}$ = 0.14). Further, although there were no group differences on the first trial of the PM clapping task (similar performance without prompting, as well as similar performance improvement for all groups after being prompted), both ASD groups performed significantly worse than the NT group (who significantly improved their performance from the first to the second unprompted trial) on the second unprompted trial. No significant group differences emerged for the PM reward task. The authors concluded that severely autistic children can succeed on certain event-based PM tasks if task characteristics are adjusted to their needs (i.e., rewarding circumstances, specific PM cues). Further they suggested that, although children with ASD benefitted from prompts in the individual trials of the PM clapping task, this did not positively affect their performance from trial 1 to 2 in contrast to the NT group. The authors suggested this could be explained by information processing deficits in ASD when the ability to connect and integrate information across tasks is required (Olu-Lafe et al. 2014).

Although Sheppard et al.’s (2016) study is interesting, it suffers from a major methodological issue in terms of matching procedure. Groups were not matched for age or gender, and no standardised measure was employed to equate groups for cognitive ability. Instead groups were matched only for national curriculum point scores for reading, writing, and number skills. The authors argue that “the demands of standard IQ tests such as WASI (Wechsler Abbreviated Scale of Intelligence, Wechsler 1999) or even BPVS (British Picture Vocabulary Test, Dunn et al. 2009) make this type of matching unsuitable for children with severe ASD” (pp. 6–7). However, it is counterintuitive that, if a child is not capable–because of their severe social-communication deficits–of completing standard IQ tests, they could complete an experimental task as complex as the task(s) used in this study (some of which were inherently social).

Fourth and finally, Altgassen and Koch (2014) studied the contribution of inhibitory control demands on event-based PM performance in 22 adults with ASD and 22 age- and nonverbal ability-matched NT adults. For this purpose, they used a *triple task* within-subject design. Participants completed two experimental conditions in counter-balanced order. The ongoing task involved a word categorisation paradigm (deciding which of two words belongs to the same category as a third word). The colour of all three words changed randomly every trial. The PM instruction was to press a pre-specified key whenever all three words were printed in blue font. Simultaneously, inhibitory load was manipulated using an auditory mental arithmetic task. In a “low inhibitory load” condition, participants were presented with a sequence of numbers via headphones and were required to add five to each number and state the resulting sum. In a “high inhibitory load” condition, the procedure was the same but participants had to withhold saying the sum aloud when it equalled 8 or 15. Contrary to the hypothesis of the authors, there were no between-group differences in PM in either condition (i.e., regardless of inhibitory load), or in the extent to which responses were correctly withheld in the high-inhibitory control condition. These findings are striking and suggest that event-based PM is not impaired in ASD even when demands on executive resources associated with successful performance are high. However, in *both* inhibitory load conditions, performance on the ongoing *categorisation* task itself was significantly superior in the comparison group than in the ASD group ($\eta _{p}^{2}$ = 0.18). As such, it remains a possibility that participants with ASD were allocating relatively more of their cognitive resources to completion of the PM task and the ongoing *inhibition* task than were comparison participants. Therefore, it may be that participants with ASD were using alternative, compensatory strategies to succeed on the PM component of the task at the expense of performance on the ongoing activity. Problems with multimodal integration (Baum et al. 2015) in this particular triple-task design might have led individuals with ASD to focus more on one of the three tasks. That is, it might be that when attentional demands of the environment are high, people with ASD may need to prioritise carrying out the planned PM action *at the expense of* other activities to an extent that NT individuals do not. Either way, the fact that participants with ASD in Altgassen and Koch’s study performed comparably to NT individuals on a PM task that had very high executive demands suggests that this ability cannot be grossly impaired in adults with this disorder.

Overall, with the exception of study of Altgassen and Koch (2014), the studies discussed above, suggest that event-based PM is impaired in ASD contrary to the initial landmark studies reviewed. However, based on the critical analysis of the studies’ methodology, such a conclusion should be drawn tentatively at best. Below we will discuss a final set of studies exploring PM in ASD in more naturalistic settings.

### “Real-Life/Naturalistic” Studies of Time- and Event-Based PM

To explore both time- and event-based PM in a naturalistic setting Altgassen et al. (2012) tested 25 adults with ASD and 25 NT participants matched for chronological age and intellectual abilities. The ongoing task consisted of preparing breakfast (using props) for four people following a simple set of rules. Two time-based (taking the tea bag out of the tea after 3 min; putting butter on the table 6 min before guests arrive) and two event-based (preparing tea immediately after kettle went off, which was indicated by the kettle changing colour; turning off the egg cooker when it beeped) PM components were embedded the breakfast preparation routine. The aim of this complex paradigm was to mirror real life PM demands. In addition, participants also completed a standard measure of event-based PM (Red Pencil Test; Salthouse et al. 2004), in which participants were asked to repeat the words ‘red pencil’ whenever the experimenter said ‘red pencil’ throughout the experimental session (which happened twice). Based on previous research, the authors predicted that only time-based PM would be diminished in the ASD group. In fact, however, participants with ASD showed diminished performance (i.e., more failures to complete the PM action) across all PM tasks. In relation to the time-based PM components of the breakfast task, the ASD group also monitored the time less often than the NT group.

The authors concluded that all aspects of PM ability are impaired *under real-life conditions* in individuals with ASD. Although this conclusion may well be accurate, whether or not we can be certain of this from the data in this study is debateable. As the study required participants to carry out both time- and event-based PM tasks within the same ongoing task (breakfast preparation), difficulties with time-based PM could have potentially carried over to the event-based PM performance in the ASD group. Thus, it may well be that ASD participants might not have been impaired in event-based PM in a real-life setting if they were not simultaneously having to carry out time-based PM. Moreover, the ongoing task itself appeared to be significantly more challenging for the ASD group than for the comparison group, as indicated by significantly worse overall ongoing task completion, as well as significantly less rule adherence and less efficient performance throughout the experiment among ASD participants. Finally, it is highly problematic that memory for the PM instruction was not checked after task completion. This is crucial, because there was a minimum delay of 15 min between encoding the PM instruction and the beginning of the experimental task, which might have promoted forgetting of the task rules.

Two other studies aimed to explore PM in ASD in a more “real-life” setting. Both used the Virtual Week paradigm (see Rendell and Henry 2009 for full details of the paradigm). The Virtual Week is a computerised single player board game that simulates 5–7 days of a week. Depending on the version of the task, the day and time of the virtual day are either always visible, or appear only after a particular keyboard key is pressed by the participant. The player rolls a die and moves a token around a board of 121 squares that represents one virtual day. On each virtual day, the player will pass ten squares, which require them to pick up an action card. Each action card poses a question about an activity for that time of the day that the participant has to make a decision about (e.g., choosing between three options for that day’s breakfast). Importantly, each choice determines whether the player has to roll a specific, an odd, or any number with their die on their next move to be allowed to continue moving their token around the board (which is revealed to them after selecting an activity option). Participants are unable to move on and have to repeat rolling the die until they have rolled the specific die number required. Hence the demands of rolling the die, moving the token around the board and making decisions about the activities to participate in, serve as the ongoing activity. Due to this board game nature of the task, the Virtual Week does not provide a measure of ongoing task performance per se, unlike more laboratory PM tasks. Additionally, each day, a total of four time- and four event-based PM tasks have to be carried out, in addition to the ten activity-related decisions that are inherent to the game. The event-based PM tasks have to be carried when a particular event occurs (as indicated by the action cards; e.g., take medication at breakfast would be triggered by the breakfast card). The time-based PM tasks have to be carried out at a set time of the virtual day (e.g., phone the plumber at 5 pm). Some PM tasks require execution on a regular basis whereas others are irregular. The regular ones are the same on each day of the Virtual Week (two time-based, two event-based). The irregular ones are one-off tasks that are instructed at the beginning of, or during, a new virtual day (two time-based, two event-based). Thus, the retrospective memory load for the irregular PM tasks is higher compared to the regular ones. At the appropriate moment to execute a PM task, the participant has to press a “perform task” button to bring up a list of possible tasks, and then choose the correct one.

One of the two studies which have used this Virtual Week paradigm with individuals with ASD was that of Henry et al. (2014). This study explored how differential levels of task absorption (i.e., the level of engagement in the ongoing task) affected PM in 30 children with ASD and 30 NT children matched for age and IQ. Tasks in the Virtual Week were adjusted to reflect children’s everyday life. Further, to reduce/eliminate time-monitoring demands, the time of each virtual day was always present in the centre of the screen. Participants completed the Virtual Week game under two conditions (each lasting three virtual days) in counterbalanced order. In the *high task absorption* condition, participants could only continue moving their token around the board if they rolled a specific number after an event card (see description above), whereas in the *low absorption condition*, the outcome of the next die roll was not restricted. The authors predicted that high task absorption may lead to greater PM impairment in the ASD group. A 2 (Group: ASD/NT) × 2 (Task absorption: low/high) × 2 (Type of PM task: event-based/time-based) × 2 (Regularity: regular/irregular) mixed ANOVA was conducted to explore effects on PM performance. Most importantly, a significant Group × PM task interaction ($\eta _{p}^{2}$ = 0.21) was found indicating that the ASD group was only impaired in time-based but not event-based PM, which ties in with the consistent pattern found by the landmark (tightly controlled) studies summarised above. Contrary to author predictions, high task absorption did not affect the ASD group to a greater extent. Further, the Group × Regularity interaction approached significance (p = .06, $\eta _{p}^{2}$ = 0.06). The authors broke this marginally non-significant interaction down using post-hoc t-tests. These *t* tests showed no performance difference between regular and irregular PM tasks within the ASD group ($\eta _{p}^{2}$ = 0.02), whereas NT individuals performed slightly better on regular PM tasks (p = .04, $\eta _{p}^{2}$ = 0.07). However, in comparison to the NT group, participants with ASD were still less accurate on both regular ($\eta _{p}^{2}$ = 0.21) and irregular ($\eta _{p}^{2}$ = 0.16) PM tasks. Therefore, authors concluded that PM difficulties in children with ASD are not a result of retrospective memory processes. Instead, they suggested that a monitoring deficit might underlie their PM deficits as time-based PM requires more self-initiated monitoring processes.

Another study which used the Virtual Week set-up was that of Kretschmer et al. (2014). Rather than focusing on the degree of absorption (which presumably affects retrieval of the original intention to carry out an action), Kretschmer et al. (2014) used this paradigm to investigate the effects of different encoding strategies on PM in a sample of 27 adults with ASD and 27 NT adults matched for age, verbal, and non-verbal abilities. The Virtual Week setup and tasks were as described above, but participants had to complete only three virtual days, and needed to press a specific keyboard key to display the time of the day. PM encoding was compared across two between-subject conditions, the first being a ‘standard’ condition and the second requiring implementation intentions, which is an encoding strategy that requires to form ‘if-then’ statements; that is, creating a specific situation when, where, and how to perform one’s intention (Gollwitzer 1999). Implementation intentions have been shown to improve PM in NT studies (Chen et al. 2015) and are thought to support episodic future thinking (Atance and O’Neill 2001). Hence, this was the first study exploring strategies to enhance PM in ASD. Specifically, in the implementation intention condition, participants had to form such an ‘if-then’ statement for each irregular PM task instruction of that day after the instructions had been presented on-screen (e.g., say out-loud, “when it is 5 p.m., then I will press the ‘perform task’ button and select ‘phone the plumber’”; Kretschmer et al. (2014), p. 3112). The logic here was that forcing participants with ASD to form implementation intentions would support their PM and, thus, raise their performance level to one commensurate with that among NT participants. No additional instructions were given in the standard condition. To analyse experimental task performance, the authors ran a 2 (Group: ASD/NT) × 2 (Encoding condition: standard/implementation intention) × 2 (Type of PM task: event-based/time-based) × 2 (Regularity: regular/irregular) mixed ANOVA to analyse PM performance across all three virtual days. They found a main effect of group ($\eta _{p}^{2}$ = 0.14), as well as a significant Group x Regularity ($\eta _{p}^{2}$ = 0.12) interaction. Interestingly, post-hoc test results revealed no within-group differences for NT adults, but ASD participants performed better on regular than irregular PM tasks ($\eta _{p}^{2}$ = 0.17). Because no Group × PM task interaction emerged, the authors concluded that individuals with ASD have a general deficit across *both* time- and event-based PM. The Group × Encoding condition interaction was non-significant with a small to medium effect size (p = .08, $\eta _{p}^{2}$ = 0.06). Nonetheless, the authors broke down the interaction effect. Post-hoc between-participant tests revealed that, relative to comparison participants, individuals with ASD showed diminished performance in the *standard* condition only; whereas in the implementation intentions condition, the between-group differences in performance were non-significant. Based on these results, the authors concluded that implementation intentions might present a strategy to support PM in individuals with ASD. However, a closer inspection of the results suggests that this conclusion is not entirely warranted. Kretschmer (personal communication, October 2016) provided the group means and SDs, which indicated that the ASD group only benefitted from employing implementation intentions for event-based PM [ASD: M_ImplementationIntentions_ = 0.81 (SD = 0.22), M_Standard_ = 0.62 (SD = 0.33); TD: M_ImplementationIntentions_ = 0.84 (SD = 0.22), M_Standard_ = 0.80 (SD = 0.29)]. However, rather than implementation intentions improving time-based PM performance of ASD, they instead *decreased* the performance of comparison participants (relative to the standard condition performance) [ASD: M_ImplementationIntentions_ = 0.53 (SD = 0.32), M_Standard_ = 0.49 (SD = 0.32); TD: M_ImplementationIntentions_ = 0.58 (SD = 0.25), M_Standard_ = 0.79 (SD = 0.24)]. Further, in contrast to Henry et al. (2014), the authors concluded that retrospective memory demands are important to understand PM deficits in ASD as participants only showed significant impairments in the irregular (one-off non-routine) PM tasks (p < .001, $\eta _{p}^{2}$ = 0.27), which place particularly high demands on retrospective memory. Unfortunately, the authors could not check whether participants actually remembered the irregular PM task instructions after each virtual day; although participants had to repeat the PM instruction three times aloud at the stage of encoding (which was supposed to ensure later remembering), there is no way of knowing whether the instruction was stored for the duration of the Virtual Week task. As such, retrospective memory limitations in ASD might explain entirely the group difference in the number of times irregular PM tasks were completed.

The Virtual Week is an interesting approach to study PM in ASD. The game format makes it easily accessible and it attempts to mirror everyday PM demands in several respects. However, the PM demands of the task are arguably much greater than (and of a different quality to) those in real life; participants have to remember *24* PM tasks (requiring the execution of *both* time- and event-based tasks and changing retrospective memory load) during a short period of time and without the use of any external reminders. Further, in the version used for both studies on ASD, the Virtual Week version did not offer the possibility to check whether participants actually remembered their PM tasks for each virtual day, which would have been particularly important for irregular PM tasks. However, Henry et al. (2014) pointed out that this feature is now part of the newest version of Virtual Week.

The results of the two studies that employed the Virtual Week paradigm differ in one major respect. Henry et al. (2014) found only time-based PM to be diminished in *children* with ASD, which is in line Williams et al.’s findings (2013, 2014). In contrast, Kretschmer et al. (2014) observed impairments of both time- *and* event-based PM in *adults*. This is surprising and requires further exploration. Kretschmer et al. (2014) employed a version of the Virtual Week that was equivalent to Henry et al.’s (2014) high-absorption condition. A possible explanation for the differing pattern of results could be the aforementioned retrospective memory difficulties in the ASD group. Kretschmer et al. (2014) found that the ASD group performed worse on the irregular PM tasks that posed the highest retrospective memory demand. This result may reflect the viable possibility that participants with ASD simply forgot the PM instruction more frequently. In general, the possibility that event-based PM deficits in ASD are observed only when demands on retrospective memory are high is brought into focus by the findings from a very large study of “everyday memory” by Jones et al. (2011).

Jones et al. (2011) investigated everyday memory in 94 adolescents with ASD and 55 age-, and IQ- (verbal, performance, and full scale) matched NT peers. Jones et al. used the Rivermead Behavioural Memory Test (Wilson and Baddeley 1991) to assess everyday memory across multiple subtests, three of which tested event-based PM. These sub-tests involved (a) reminding the experimenter about the location of a pen upon the occurrence of a particular verbal cue; (b) asking the experimenter a question when an alarm went off; and (c) remembering to pick up an envelope before walking a route as demonstrated by the experimenter. The ASD group achieved a significantly lower PM composite score (across the three subtests) than the comparison group, indicating significant event-based PM impairments in this very large sample of ASD participants. However, Williams et al. (2013) noted that Jones et al.’s analysis included participants who had failed to remember the PM task instruction at all. When the data from Jones et al. (2011) were re-analysed excluding participants who completely failed to recall the PM instruction, there was no hint of any between-group differences in PM task performance (Williams et al. 2013, p. 1564). This re-analysis underscores the importance of controlling for retrospective memory demands when drawing conclusions about PM ability in ASD.

## Discussion

### Why is Time-Based PM Impaired in ASD?

The evidence from the meta-analysis presented in Part 1, as well as our review of the evidence in Part 2, suggest strongly that time-based PM in ASD is impaired. Of course, the challenge remains to explore the underlying reasons for this impairment. Several studies have explored the underlying cognitive correlates of time-based PM in ASD (see Table 4).^2^ Although these correlation analyses offer some indication about the underlying cause of time-based PM impairments in ASD, they are far from conclusive. In general, time-based PM performance appears to be related to executive functioning processes in ASD. Given that time-based PM requires self-initiated (rather than cued) retrieval of intentions, it is unsurprising that executive processes might be related to task performance, since self-initiated retrieval of information from memory is considered to place a high demand on executive functioning (e.g. McDaniel and Einstein 2007). It may be, therefore, that well-established difficulties with aspects of executive functioning in ASD underpin time-based PM deficits in ASD.

**Table 4:** Overview of PM correlates

		Clock checks	DEX	General cognitive ability	Inhibition	Switching/cognitive flexibility	Theory of mind	Verbal processing efficiency	Verbal fluency/semantic switching	Working memory	ABAS	Autism severity	PRMQ PM-scale
Time-based	ASD	r = .73^*,a^ r = .80^*,e^		FSIQ r = .51^*,f^	Stroop r = .47^*,f^		Animations r = − .42^*,e^	r = .41^i^	r = .30^f^		r = .57^*,f^	Not studied	
	NT	r = .86^*,a^ r = .47^*,e^				WCST r = .32^†,e^			r = .54^*,f^	Visual complex span r = .39^g^ Verbal storage span r = − .38^g^	r = .45^*,f^		
	Both	r = .82^*,a^	r = − .38^*,d^			TMT r = −.38^*,d^				Digit ordering r = .33^d^			
Event-based	ASD	N/A	r = − .48^*,b^	NVIQ r = .52^*,h^		DCCS r = .34^h^	Not studied	r = .45^*,g^		Verbal complex span r = − .36^g^ Verbal storage span r = − .52^*,g^ Block span r = .45^*,g^		CARS r = −.34^†,i^ ADOS-SC r = −.37^*,c^ ADOS-R r = −.21^*,c^	
	NT	N/A	r = − .31^b^	NVIQ r = .34^i^	Stroop r = .56^*,h^	TMT r = .32^f^	Not studied		r = .43^*,f^	Visual complex span r = −.30^g^ Visual storage span r = −50^*,g^	r = .30^f^		r = .43^†,g^
	Both		r = − .36^*,b^										

*ABAS* Adaptive Behavior Assessment Scale, *DEX* Dysexecutive Questionnaire, *PRMQ* Prospective Retrospective Memory Questionnaire, *FSIQ* Full scale IQ, *NVIQ* Non-verbal IQ, *DCCS* Dimensional Change Card Sort task, *TMT* Trail Making Task; *WSCT* Wisconsin Card Sorting Task, *CARS* Childhood Autism Rating Scale, *ADOS-SC* Autism Diagnostic Observation Schedule social communication, *ADOS-R* Autism Diagnostic Observation Schedule repetitive behaviour, *N/A* not applicable

*Significant p < .05

†Marginal significant p < .09; marginal and non-significant correlations are only included in the table if at least of moderate size (r ≥ .30)

^a^Altgassen et al. (2009)

^b^Altgassen et al. (2010)

^c^Jones et al. (2011): FSIQ partialled out

^d^Altgassen et al. (2012)

^e^Williams et al. (2013): ongoing task performance partialled out

^f^Henry et al. (2014)

^g^Williams et al. (2014): ongoing task performance partialled out

^h^Yi et al. (2014)

^i^Sheppard et al. (2016)

No correlations were assessed in Altgassen et al. (2014), Brandimonte et al. (2011), and Kretschmer et al. (2014)

An alternative possibility is that difficulties with representing mental states (i.e. theory of mind) in ASD make it particularly difficult for people with ASD to introspect/retrieve their own intentions (e.g., Williams and Happé 2010). Given the link between PM and theory of mind in NT individuals (e.g. Ford et al. 2012), this could explain time-based PM deficits in ASD. Indeed, the association found by Williams et al. (2013) between time-based PM and theory of mind supports this possibility. The idea is that theory of mind is relevant for mental self-projection, i.e. the ability to shift one’s perspective from the immediate present to alternative perspectives (Buckner and Carroll 2007), which overlaps with episodic future thinking processes. Hence NT individuals might not only think more frequently about their delayed intentions but also, if they do, mentally simulate their execution, strengthening relevant context associations for later PM retrieval. Therefore, if individuals with ASD have difficulties with theory of mind and with generating future episodic representations as a result of diminished self-projection ability (see Lind et al. 2014, for relevant evidence), this would be expected to contribute to poor time-based PM performance (see Williams et al. 2013, for a discussion of this possibility).

One final possibility to consider is that time-based PM deficits in ASD may result from difficulties with time perception (see Allman and Meck 2012). However, neither time-estimation (Shah et al. 2016) nor reproduction (Wallace and Happé 2008) of time units broadly similar to those involved in time-based PM tasks (up to 49 s) appear to be impaired in ASD. Data from studies of time-based PM are also informative. In five out of the aforementioned six studies of time-based PM, participants had to monitor the elapsed time during the experiment (by pressing a keyboard key to bring up a clock), which depends on time perception. Two of these studies did not find any indication of between-group differences in time monitoring despite finding significant time-based PM impairments (Williams et al. 2013, 2014), whereas two reported significantly fewer clock-checks in ASD (Altgassen et al. 2009, 2012). However, in the studies by Altgassen et al. memory for task instructions was not assessed, meaning that between-group differences in time-monitoring might be attributable to a failure to recall that the time even needed to be checked. Overall, the existing evidence regarding time perception in ASD does not provide strong support for the hypothesis that impaired time monitoring is the major cause of time-based PM problems in ASD, although future research should consider this explicitly.

Taken together, it is clear that there are multiple potential causes of the evident time-based PM impairment in ASD. Although studies of the cognitive correlates of this impairment are potentially informative, Table 4 illustrates the “patchwork” nature of results, as well as the fact that almost no correlate has been studied systematically across studies. Future studies might consider focussing on the key candidate underlying causes of this PM impairment and conduct systematic investigations of those.

### Is Event-Based PM Really Impaired in ASD?

Although the conclusions drawn in parts 1 and 2 of this paper were remarkably consistent in suggesting a large impairment of time-based PM in ASD, this consistency was not evident with regard to event-based PM. Although the meta-analysis presented in Part 1 provided evidence of a subtle (statistically small) impairment of event-based PM in ASD, the review presented in Part 2 questions the validity of the evidence from the meta-analysis. Eleven studies have investigated event-based PM among individuals with ASD. Five of the studies found an impairment in ASD, while six did not. There is no clear pattern with regard to age; half of the studies found an impairment in their adult/children samples while the other half did not. However, if one only considers the studies that fulfilled the aforementioned methodological guidelines necessary for studying PM in ASD, evidence points toward unimpaired event-based PM in ASD.

A careful consideration of the studies of event-based PM that were included in the meta-analysis suggest that the methodological rigour of several of the studies was not sufficiently high to draw strong conclusions from the meta-analytic data. That is, although the *results* from *across* all studies of event-based PM in ASD suggest a subtle impairment in ASD, the methods that produced those results may not be valid and/or reliable enough to allow a firm conclusion from the results to be drawn. There are also a priori reasons to hypothesise that event-based PM should be unimpaired (despite impaired time-based PM) in ASD. The profile of strengths and weaknesses in *retrospective* memory in ASD suggests that this ability is impaired only when tests of memory are uncued/unstructured (see Boucher et al. 2012). For example, in tests of free recall, which require *self-initiated* retrieval of information from long-term memory (paralleling the demands of *time*-based PM), adults and children with ASD tend to show diminished performance. In contrast, in tests of cued recall or recognition (where a cue/the context for retrieval is provided, paralleling the demands of *event*-based PM), adults and children with ASD tend to show *un*diminished performance. These findings have led to suggestions that only unstructured/unsupported cognitive/memory tasks will be impaired in ASD (the “task support hypothesis”; e.g., Bowler et al. 2004). Our analysis in Part 2 of this paper is in line with this hypothesis.

## Future Directions

PM impairments can seriously impact an individual’s everyday life and independent functioning. This review indicates that time-based PM is challenging for individuals with ASD, which is consistent with self-reports (Williams et al. 2014). Event-based PM problems might also represent a challenge for people with ASD in everyday life. However, this review indicates that evidence for an event-based PM impairment in ASD is mixed, at best. Methodological limitations with several of the existing studies of event-based PM prevent firm conclusions about the extent to which this ability is impaired in ASD. Further, well-controlled studies need to investigate event-based PM systematically and taking into consideration the methodological guidelines outlined above.

In practice, PM impairments in ASD may result in reduced autonomy and greater dependency on carers, parents, or partners to support daily activities and time-management. For instance, PM problems might lead to forgetting to pay their mobile phone bill, pick up a parcel from the post-office, or to attend a medical appointment (Blomqvist et al. 2015). Equally, PM impairments could negatively impact employment opportunities for individuals with ASD (employers might understandably perceive a person with diminished PM as unreliable, because they forget to complete assignments, meet deadlines, or pass on important message), which may contribute to low rates of full-time employment among individuals with ASD (Howlin and Moss 2012).

Given the well-established, large diminution of time-based PM in ASD, it will be important to develop training strategies to support/enhance this ability in order to promote greater functional independence. This requires to fill in the gaps in our understanding of how individuals with ASD engage in time-based prospective remembering. To this end, a systematic exploration of cognitive mechanisms that underpin PM performance in ASD is necessary. Conversely, finding strategies that may improve time-based PM could also reveal underlying causes of its impairment in ASD. If, for example, efforts to improve executive functioning (or episodic future thinking or theory of mind) were found to improve time-based PM in individuals with ASD, this would suggest that executive dysfunction is a key contributory factor to diminished time-based PM in ASD. Another approach could be to test whether manipulations that lead to PM improvement in older NT adults might have a similar beneficial effect in ASD (see Hering et al. 2014 for a review). For instance, using rewards to increase a person’s motivation has previously been shown to have positive effects on PM performance in NT samples (see Walter and Meier 2014 for a review). However, causes for PM problems in ASD may not necessarily be the same as in healthy ageing. Hence, transfer of seemingly useful PM strategies (e.g. implementation intentions, active cue monitoring, time-checking training, increasing motivation) in NT individuals may not lead to a reliable PM improvement in ASD. It seems clear that PM research in ASD is in its infancy, relatively-speaking, and that there are as more unanswered questions than questions answered. This review provides suggestions and guidelines for future research, which we hope will be useful for clinicians and researchers alike when considering this ability in ASD.

## Footnotes

1 In four studies, a decision had to be made about which of the multiple effect sizes reported should be included in the meta-analysis. Our decisions were based entirely on the rationale for the study and/or study hypotheses, and not on study results. Importantly, taking alternative decisions would not have changed the results of the meta-analysis substantively. Moreover, for completeness, the effect sizes that were not included in the meta-analysis are displayed in the forest plots (Figs. 2, 3). In Sheppard et al. (2016), who tested a mildly and severely autistic group of children, the effect size from the group contrast between the severely autistic vs. neurotypical children was used in line with Sheppard et al.‘s (2016) hypothesis that only severely autistic children would show a PM impairment. In Yi et al. (2014), who included two comparison groups (one matched for chronological age with the ASD group, and one matched for mental age with the ASD group) in their study, the contrast between ASD and the ability-matched NT group was used as the age-matched NT group performed at absolute ceiling on the PM task making valid comparison with the ASD group impossible. For Altgassen et al. (2012) who used a standard measure of PM in addition to a naturalistic PM task, the standardised mean differences for the two contrasts (which were very similar) were averaged into a composite effect size. Finally, Kretschmer et al. (2014) manipulated a between-subject factor that aimed to improve PM using an encoding strategy in comparison to a standard no strategy condition. As this meta-analysis aimed to estimate the extent of true PM impairment in ASD, inclusion of the strategy contrast in the analysis would bias the weighted effect size. Hence, we used the contrast where participants performed the PM task under the no-strategy condition.

2 Table 4 also outlines the cognitive correlates of event-based PM. However, given that a number of the studies included had methodological drawbacks, even where significant relationships with potential cognitive underpinnings were found, these need to be interpreted with caution.
